# Serum Metabolomics and Ionomics Analysis of Hoof-Deformed Cows Based on LC-MS/MS and ICP-OES/MS

**DOI:** 10.3390/ani13091440

**Published:** 2023-04-23

**Authors:** Chaoyang Deng, Yang Yue, Hefei Zhang, Meng Liu, Yansong Ge, Enshuang Xu, Jiasan Zheng

**Affiliations:** College of Animal Science and Veterinary Medicine, Heilongjiang Bayi Agricultural University, Daqing 163000, China

**Keywords:** dairy cow, hoof deformity, metabolomics, ionomics, joint analysis

## Abstract

**Simple Summary:**

In recent years, due to the increasing scale of pasture, the incidence of hoof disease is increasing and hoof deformation is one of the common hoof diseases in dairy cows. In order to explore the metabolic and ionic changes of hoof-deformed dairy cows, LC-MS/MS and ICP-OES/MS techniques were used to analyze the serum samples of dairy cows. As a result, 127 metabolites were screened by metabolomics, and 28 metabolic pathways were enriched by KEGG metabolic pathway enrichment analysis, including nine metabolic pathways. The results of ion omics showed that 13 kinds of ions such as K, Li and Pb were up-regulated, while 18 kinds of ions such as Al, Cu and Sb were down-regulated. The integrated analysis of metabolomics and ion omics found that potassium ions were positively correlated with L-tyrosine and L-proline, positively correlated with thiamine and negatively correlated with L-valine. Sodium ions were positively correlated with L-valine and negatively correlated with α-D-glucose. The aim of this study was to investigate the ionic and metabolic changes in the organism after hoof deformation in dairy cows.

**Abstract:**

In order to explore the metabolic and ionic changes of hoof-deformed cows, the serum samples of 10 healthy cows (group C) and 10 hoof-deformed cows (group T) were analyzed by LC-MS/MS and ICP-OES/MS. The pathway enrichment of differential metabolites was analyzed by screening and identifying differential metabolites and ions and using a bioinformatics method. The integration of metabolomics and ionics was analyzed with ggplot2 software in R language, and verified by MRM target metabolomics. The results showed that 127 metabolites were screened by metabolomics, of which 81 were up-regulated (*p* < 0.05) and 46 were down-regulated (*p* < 0.05). The results of ICP-OES/MS showed that 13 kinds of ions such as K, Li, and Pb in serum of dairy cows were up-regulated, while 18 kinds of ions such as Al, Cu and Sb were down-regulated. The integrated analysis of metabolomics and ionics found that potassium ions were positively correlated with L-tyrosine, L-proline, thiamine and L-valine. Sodium ions were positively correlated with L-valine and negatively correlated with α-D-glucose. The results of high-throughput target metabolomics showed that the contents of L-proline, L-phenylalanine and L-tryptophan in serum of dairy cows increased significantly, which was consistent with the results of non-target metabolomics. In a word, the metabolism and ion changes in dairy cows with hoof deformation were revealed by metabolomics and ionics.

## 1. Introduction

Dairy cattle hoof and limb disease is a general term for various diseases of the limbs and hooves of dairy cows, which have a great impact on the health and performance of dairy cows and are second only to mastitis and reproductive disorders [1]. Hoof deformation is one of the common hoof diseases among dairy cattle limb and hoof diseases. Hoof deformation in cows can promote the development of hoof diseases. It has been suggested [2] that the occurrence of hoof deformation is associated with various factors such as geographical location, climate, feeding management, mineral elements, disease, and blood rheology. Among metabolomics, non-targeted liquid chromatography-mass spectrometry (LC-MS) metabolomics techniques are widely used in clinical studies. Ionomics is a technique used to study the composition, distribution, and accumulation of ions in a test sample. Inductively coupled plasma mass spectrometry (ICP-MS) is the most commonly used mass spectrometry-based elemental analysis method for the simultaneous detection of multiple elements, and it can be used in conjunction with other chromatographic separation techniques for elemental valence and isotope analysis.

Recently, Zheng et al. [3] applied metabolomics techniques to hoof rot and showed that cows with hoof rot have altered carbohydrate, amino acid, lipid, and energy metabolic pathways. Zheng et al. [4] conducted a proteomics study of hoof rot in cows based on bidirectional electrophoresis mass spectrometry and identified a differentially expressed protein. Sun [5] et al. examined the plasma of cows with hoof rot using bird shotgun method proteomics and concluded that PDE proteins PGRP-L and KS-PG are potential biomarkers of hoof rot in dairy cows; these studies suggest that histological techniques have been widely used in hoof disease research in dairy cows. Sun et al. [6] analyzed the relationship between metabolism and obesity by linking metabolomics and ionomics. However, no metabolomic and ionomics studies have been reported on hoof deformation in dairy cows. In the present study, we investigated the changes in the metabolism and ionogenesis of hoof-deformed cows from metabolomic and ionomic perspectives.

## 2. Materials and Methods

### 2.1. Animals and Experimental Design

Animal experiments were conducted in accordance with the International Guidelines for Animal Biomedical Research. The experiments were supervised by the Sub-Committee on Ethics of Science and Technology, College of Animal Science and Technology, Heilongjiang Bayi Agricultural University (license number SY201909005).

The experiments were conducted on an intensive dairy farm in Heilongjiang province, housing approximately 1500 Holstein cows. The cows collected for this experiment were all early lactation cows with a TMR time of three times a day, and a TMR diet consisting of 25 kg of corn silage, 1 kg of imported grass clover, 0.5 kg of oat grass, 3.5 kg of pressed corn, 2 kg of cornmeal, 2.6 kg of soybean meal, 0.5 kg of wine lees, 1 kg of rumen-loaded soybean meal, 1 kg of whole cottonseed, 8 kg of wet beer lees, 1.8 kg of molasses, 0.3 kg of calcium fatty acid, 0.05 kg of encapsulated urea, 20 g of chromium tripyridate, 0.15 kg of sodium carbonate, sodium chloride 40 g, yeast 30 g, mold adsorbent 20 g, water 3 kg, 1% of various trace elements and vitamin premix additives 1.2 kg. Professional hoof trimming was performed twice a year. Twenty cows were selected and divided into the following two groups (n = 10 cows per group): (1) the control group (C group): healthy cows with similar age, parity, and physical condition; (2) the hoof deformation group (T group): cows with wide hooves and no other diseases. Diagnosis of cows with deformed hooves is referred to as “Control of deformed hooves and hoof diseases in dairy cows” [7].

### 2.2. Sample Collection

#### 2.2.1. Blood Sample Collection

Blood was collected from cows in the early morning before TMR. Blood was collected from the test cows via the caudal vein. The specific site for blood collection from the tail was about 10 cm from the tail root, at the mid-point depression of the junction of the 4th and 5th caudal vertebrae. After disinfection, the disposable blood collection device was held, and the right index finger controlled the depth of the needle, and the needle was stabbed vertically from the bottom to the ventral centerline of the cow’s tail for about 0.5 cm, and blood was drawn when there was blood return. A total of 10 mL of whole blood was collected from each cow, and the serum was collected after centrifugation at 3000× *g* for 10 min for subsequent analysis.

#### 2.2.2. LC-MS/MS

Transfer 100 μL serum sample to EP tube, add 400 μL extract (methanol:acetonitrile = 1:1 (v/v), containing isotope labeled internal standard mixture), vortex and mix evenly for 30 s and ultrasonic for 10 min (ice water bath). Let the sample stand at −40 °C for 1 h, then centrifuge at 4 °C and 12,000 rpm (centrifugal force of 13,800× *g*, radius of 8.6 cm) for 15 min, and take the supernatant into a sample bottle for computer detection. All samples were mixed with the same amount of supernatant to form QC samples for computer detection.

Chromatographic separation of target compounds was performed on a Waters ACQUITY UPLC BEH Amide (2.1 mm × 100 mm, 1.7 μm) liquid chromatographic column using a Vanquish (Thermo Fisher Scientific) ultraperformance liquid chromatograph.

#### 2.2.3. Determination of Each Element by ICP-OES/MS with Hydrogen Peroxide-Purified Nitric Acid System Digestion

The serum samples were thawed and shaken well. First, 0.5–1 mL of serum was put in a plastic polyester bottle. Then, 0.5 mL H_2_O_2_ (30%) and 2 drops of purified HNO_3_ (2%) were added for digestion. Purified water was added to reach a total volume of 10 mL, and samples were shaken well, allowed to stand for 1 day, and then analyzed.

The following elements were analyzed: Ca, K, Mg, Na, Fe, Sn, Be, As, Al, Cr, Mn, Co, Bi, P, Ni, Mo, B, Ag, Cu, Zn, Se, Cd, Sb, Ba, Ti, Pb, Zr, S, Li, Si, and Sr. After digestion, the sample was determined by Thermo Fisher Scientific iCAPRQ inductively coupled plasma mass spectrometer. The element-specific mass number (mass-to-charge ratio, *m*/*z*) was determined by the external standard method, and the intensity ratio of the mass spectral signal of the element to be measured to the mass spectral signal of the internal standard element was quantified to determine the concentration of the element to be measured.

#### 2.2.4. MRM Target Verification

The serum samples were thawed and mixed by vortexing for 30 s. Next, 15 µL of each sample was put into a 1.5-mL EP tube, to which 185 µL of pre-chilled (−40 °C) extraction solution (methanol:acetonitrile:water = 80:80:25 [v/v/v], containing isotopically labeled internal standard mixture) was added. Samples were mixed by vortexing for 30 s, then sonicated in an ice water bath for 15 min, and allowed to stand at −40 °C for 1 h. The samples were centrifuged at 4 °C at 13,800× *g* for 15 min, and 80 µL of supernatant was put into the LC injection bottle for UHPLC-MS/MS analysis.

The chromatographic separation of the target compounds was performed on an Agilent 1290 Infinity II series (Agilent Technologies) ultraperformance liquid chromatograph using a Waters ACQUITY UPLC BEH Amide (100 × 2.1 mm, 1.7 μm, Waters) liquid chromatographic column.

### 2.3. Statistical Analysis of Data

#### 2.3.1. LC-MS/MS Data Processing, Statistical Analysis, and Metabolic Pathway Analysis

Univariate statistical analyses included Student’s *t*-test. Multivariate statistical analyses included principal component analysis (PCA) and orthogonal partial least squares discriminant analysis (OPLS-DA). Other differential compounds were screened and identified. Hierarchical clustering analysis, correlation analysis of differential metabolites, KEGG annotation, differential metabolite pathway analysis, and regulatory network analysis of differential metabolites were performed after screening for differential metabolites.

#### 2.3.2. ICP-OES/MS Data Processing and Statistical Analysis

Univariate statistical analyses included Student’s *t*-test. Multivariate statistical analyses included PCA, stratified cluster analysis, radar plot analysis, bi-plot analysis, boxplot analysis, and receiver operating characteristic (ROC) curve analysis.

#### 2.3.3. MRM Data Processing

The final measured concentration (*C_F_*) (nM) was calculated by multiplying the calculated concentration (*C_C_*) (nM) by the dilution factor (Dil) (nM). The target metabolite concentration in the sample (*C_M_*) was calculated by multiplying *C_F_* by the final volume of the sample (*V_F_*) (μL) and dividing by the sample volume *V_S_* (μL):

$$C_{M}\left[\mathrm{nM}\right]=\frac{C_{F}\left[\mathrm{nM}\right]\cdot V_{F}\left[\mu L\right]}{V_{S}\left[\mu L\right]}.$$

## 3. Results

### 3.1. LC-MS Metabolomics Results

#### 3.1.1. Multivariate Statistical Analysis

**Figure 1:**
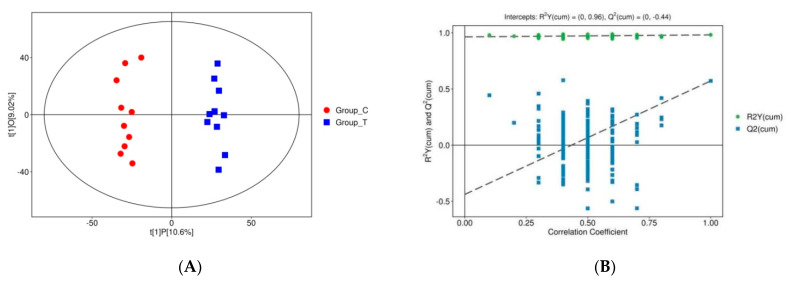
(**A**) Scatter plot of the OPLS-DA models. (**B**) Replacement test results of the OPLS-DA models. Note: The horizontal axis in the plots of group C (red) and group T (blue) in (**A**) indicates the replacement retention of the replacement test (the proportion that is consistent with the order of the Y variables of the original model; the point at which the replacement retention is equal to 1 is at the R2Y and Q2 values of the original model). The vertical axis in (**B**) indicates the values of R2Y or Q2. The green dots indicate the R2Y values obtained from the replacement test, the blue square dots indicate the Q2 values obtained from the replacement test, and the two dashed lines indicate the regression lines of R2Y and Q2.

After PCA analysis, OPLS-DA was conducted to eliminate noisy information that was not relevant to the classification (Figure 1A). Each point in the figure represents a sample, with red representing the healthy group and blue representing the disease group. The results showed that these two groups were significantly separated, largely within the 95% confidence interval. The results of the replacement test of the OPLS-DA model for the disease group to the healthy group are shown in Figure 1B. The Q2 values of the random model of the replacement test were all smaller than those of the original model; the intercept between the regression line of Q2 and the vertical axis was less than zero; and the Q2 value of the random model gradually decreased as the retention of the replacement gradually decreased and the proportion of the replaced Y variables increased. This indicates that the original model had good robustness and there was no overfitting.

#### 3.1.2. Screening and Identification of Differential Metabolites

**Figure 2:**
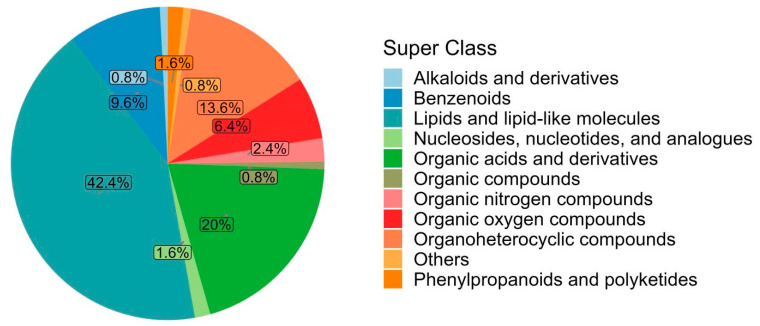
Metabolite classification. The different color blocks in the graph indicate different taxonomic categories, and the percentages indicate the percentage of metabolites belonging to that type out of the number of all identified metabolites.

The criteria used in the present study were *p* ≤ 0.05 and VIP > 1. A total of 127 differential metabolites were characterized; 81 were significantly upregulated and 46 were significantly downregulated in group C compared with group T (Table 1). The types of differential metabolites were analyzed; the categories and percentages are presented as pie charts (Figure 2). The main types of differential metabolites were lipids and lipid molecules (accounting for 42.4%), organic acids and their derivatives (accounting for 20.0%), organic heterocyclic compounds (accounting for 13.6%), benzene ring compounds, alkaloids and derivatives, nucleosides, nucleotides and analogues, and sugar polyketones.

#### 3.1.3. Hierarchical Clustering Analysis of Differential Metabolites

**Figure 3:**
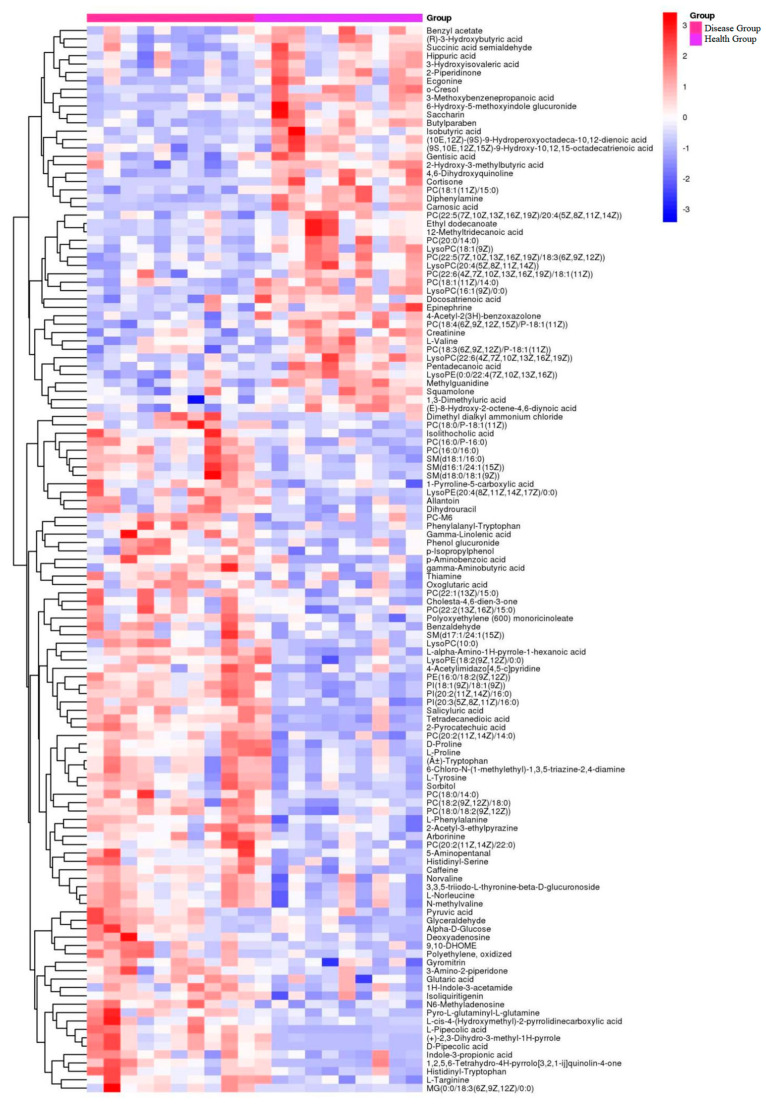
Heatmap of differential metabolites in sera of group T and group C. The horizontal coordinates in the figure represent different cows, the vertical coordinates represent the differential metabolites, and the colors represent the relative levels of metabolites, where red indicates high content and blue indicates low content.

Hierarchical clustering analysis was performed to screen differential metabolites. A Euclidean distance matrix was calculated for quantitative values of each group of differential metabolites, and the differential metabolites were clustered using a complete chain method. They are presented in a heatmap (Figure 3). The hierarchical clustering analysis showed that the levels of the differential metabolites were significantly different between experimental and control groups.

#### 3.1.4. Metabolic Pathway Enrichment Analysis of Differential Metabolites

**Figure 4:**
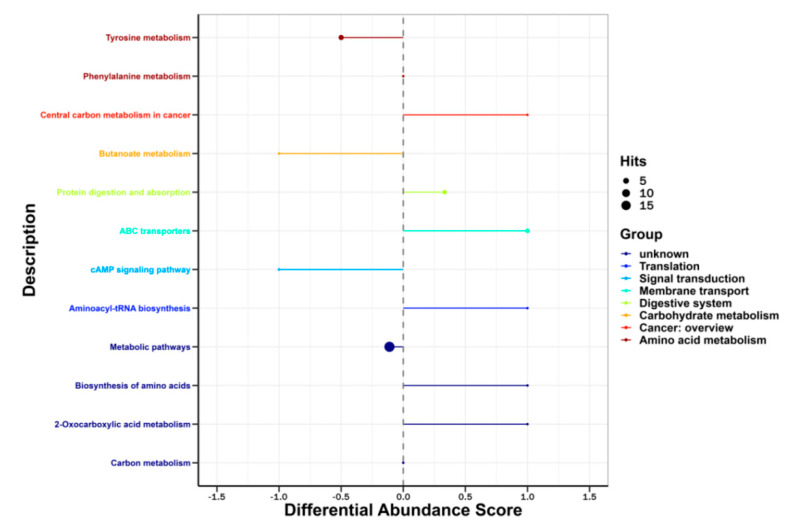
DAS diagram of metabolic pathway analysis. The horizontal axis shows the differential abundance score, the vertical axis shows the metabolic pathways, the size of the dot in the graph represents the number of differential metabolites involved in that pathway, and different colors represent the metabolic pathway categories.

**Figure 5:**
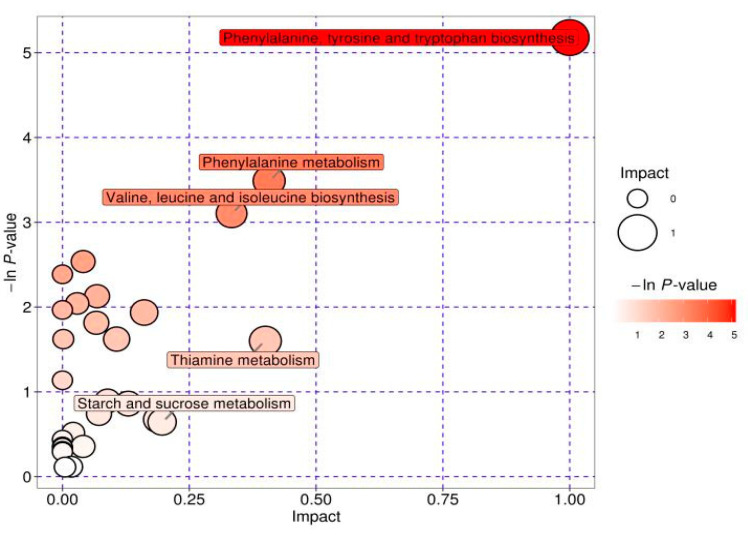
Bubble diagram of the metabolic pathway analysis results. Each bubble in the bubble diagram represents a metabolic pathway. The size of the horizontal axis where the bubble is located indicates the influence of the path in the topological analysis; the larger the bubble, the greater the influence. The vertical axis and the color of the bubble indicate the *p*-value of the enrichment analysis (taking the negative natural logarithm, i.e., −ln(*p*)); the darker the color, the smaller the *p*-value and the more significant the enrichment.

All the screened differential metabolites were mapped against the KEGG database and analyzed for metabolic pathways (Figure 4). The enrichment analysis of the matched metabolic pathways resulted in a total of 28 matches (Figure 5). The pathway enrichment analysis suggested that the main metabolic pathways that were altered in deformed cows were tyrosine metabolism, phenylalanine, tyrosine, and tryptophan biosynthesis, thiamine metabolism, valine, leucine, and isoleucine biosynthesis, starch and sucrose metabolism, pyruvate metabolism, glycolysis, and gluconeogenesis. Nine differential metabolites were involved in the above major metabolic pathways (Table 2).

### 3.2. ICP-OES/MS Ionomics Results

**Figure 6:**
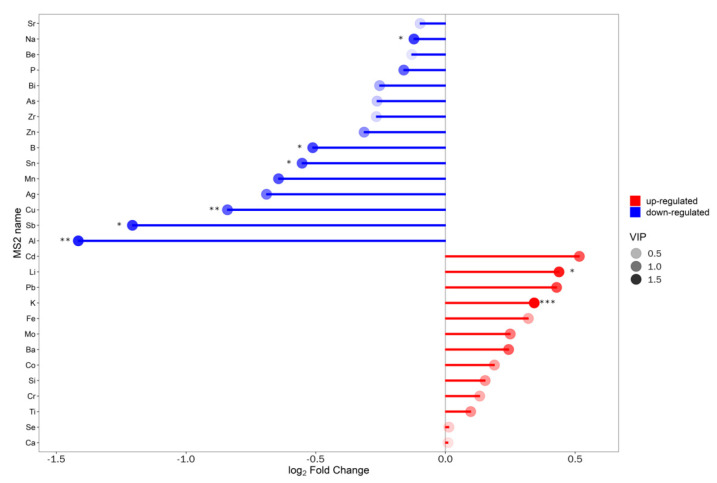
Matchstick plot of differential ions in serum of group T (disease group) vs. group C (healthy group). The horizontal axis shows the log-transformed fold change values. Red represents upregulation, blue represents downregulation, and dot color shades represent the VIP values.

**Figure 7:**
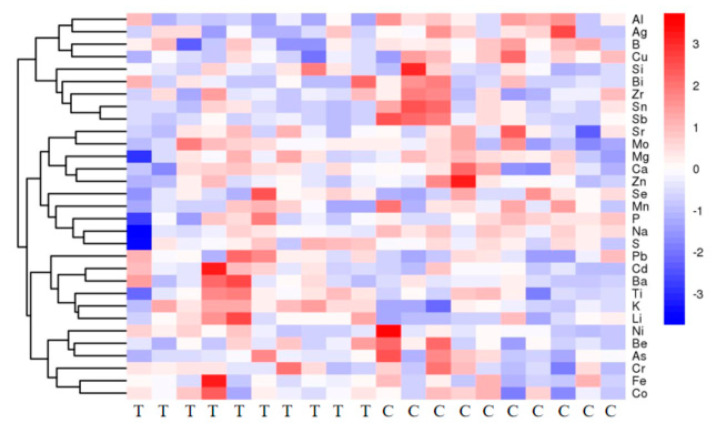
Heatmap of hierarchical clustering analysis of differential ions in sera of group T and group C. The horizontal coordinates in the figure represent different cows, the vertical coordinates represent ions, and the colors represent the relative levels of the ions, with red indicating high content and blue indicating low content.

The ICP-OES/MS results showed a significant upregulation of 13 ions and a significant downregulation of 18 ions (Table 3). Matchstick plot analysis was performed for the screened differential ions (Figure 6). The quantitative values of ions were calculated as a Euclidean distance matrix to cluster the metabolites by the complete chain method. The results are presented as a heatmap (Figure 7).

### 3.3. Joint Analysis Results

**Figure 8:**
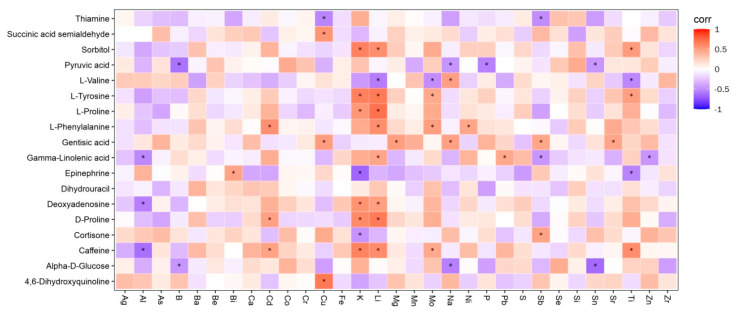
Association of differential metabolites with ions. The horizontal coordinates show the ion names and the vertical coordinates show the names of the differential metabolites. Red indicates a positive correlation, blue indicates a negative correlation, and * represents statistical significance.

Combined analysis of serum metabolomics and ionomics results was conducted using the ggplot2 package (version 3.3.2) in R (version 3.6.3). K ions were positively correlated with L-tyrosine and L-proline, positively correlated with thiamine, and negatively correlated with L-valine; Na ions were positively correlated with L-valine and negatively correlated with α-D-glucose; and a variety of ions were significantly correlated with the differential metabolites (Figure 8).

### 3.4. MRM Validation Results

L-Proline, L-Phenylalanine, and L-Tryptophan were selected for experimental validation. The results in Table 4 show significantly higher levels of the three amino acids, consistent with the non-targeted metabolomics results.

## 4. Discussion

### 4.1. Analysis of Differential Serum Metabolites in Cows with Hoof Deformities

Between 60% and 85% of circulating glucose in the mammary glands of ruminants is used for lactose synthesis [8]. The lactose production curve is similar to the lactation curve, with glucose uptake by the mammary gland being highest during peak lactation [9]. However, in high-producing cows, glucose metabolic pathways in the body are prioritized to meet the cow’s lactation requirements [10]. Hoof deformation can directly affect growth and lead to a decrease in milk production. In the present study, α-D-glucose levels were significantly higher in group T, which may be explained by the fact that lactose is mostly used to produce α-D-glucose through the TCA cycle. In addition, the process of lactation in cows requires a large amount of energy, which can lead to the large-scale conversion of body fat into fatty acids, which are further used to produce acetyl Co-A, which enters the TCA cycle to supply the body with energy [11]. The elevated plasma α-D-glucose levels in cows of group T in the present study may be related to the activation of metabolic pathways such as starch and sucrose metabolic pathways, glycolysis, or gluconeogenesis during hoof deformation.

Amino acids are substrates for protein synthesis and can also be involved in metabolism as bioactive molecules [12]. The highest enrichment in this study was in the biosynthetic metabolic pathway of phenylalanine, tyrosine, and tryptophan; the main differential metabolites within this pathway were L-phenylalanine and L-tyrosine. In the phenylalanine metabolic pathway, L-phenylalanine is used to generate L-tyrosine in the presence of phenylalanine 4-monooxygenase; therefore, the serum levels of both L-phenylalanine and L-tyrosine were significantly elevated in the T group of cows, demonstrating that the biosynthesis of phenylalanine, tyrosine, and tryptophan and phenylalanine metabolism were activated in cows during the occurrence of hoof deformation. L-valine is an important nutrient for cows and is closely related to production performance [13]. Valine is a glucose-producing amino acid which is mainly used in the synthesis of proteins and provides energy for the body, while its product glutamine and the intermediate product alanine can be used for gluconeogenesis through the alanine-glucose cycle to maintain blood glucose concentrations [14,15]. Therefore, the biosynthesis pathways of valine, leucine, and isoleucine are activated in cows with hoof deformation, and their products, including L-valine, undergo gluconeogenesis to provide energy for hoof-deformed cows. β-Alanine is the only β type amino acid found in nature and is the limiting amino acid for myostatin synthesis, which can increase carnosine content in muscle, which can enhance the body’s sports performance, anti-fatigue, anti-oxidation, and enhance the buffering capacity of muscle [16]. In the present study, the serum level of dihydroxyuracil was significantly higher in group T cows, which may be due to the activation of the β-alanine metabolic pathway after hoof deformation, and the production of dihydroxyuracil can consume a large amount of β-alanine and reduce the locomotor performance of the body.

The main function of thiamine is to participate in energy metabolism, and the amount required is directly related to energy intake. Therefore, when thiamine metabolism is abnormal in cows, glucose metabolism is also impaired. The rumen of ruminants absorbs free thiamine, but does not absorb thiamine in the bound state or inside microorganisms [17]. In the present study, the hoof-deformed cows showed a significant increase in thiamine levels due to enhanced thiamine metabolism and consequent changes in glucose metabolism.

### 4.2. Analysis of Differential Ions in Serum of Cows with Hoof Deformities

Mineral elements are essential for the maintenance of limb and hoof health in cows [18]. Ca and P are important components in bones and hoof keratin, and an imbalance in the Ca/P ratio leads to Ca deficiency in bovine bones, which easily causes severe softening and even deformation of the hoof shell keratin [19]. In the present study, the serum Ca ion content was higher and the P ion content was lower in group T, indicating that an imbalance of the Ca/P ratio may cause abnormal bone development and hoof deformation in cows. Iron is an important component of many enzymes and plays an important role in intracellular biological oxidation, but excess iron in the body will inhibit the body’s absorption, transport and utilization of zinc and copper, resulting in a decrease in the content of zinc and copper in the body, thus affecting the formation and renewal of hoof keratin in cows and leading to various hoof diseases [20]. Serum manganese and zinc levels were reduced in hoof-deformed cows in this experiment. Zinc deficiency during growth can cause limb curvature, swelling of the hock joints, slow growth and development, hindered hoof keratin renewal, incomplete keratinization of limbs, and hoof sole or lateral cleft (vertical cleft), leading to increased hoof disease [21]. Manganese can promote the synthesis of acidic mucopolysaccharide in the bone matrix and cartilage, which is used by the body to synthesize collagen and cartilage; manganese deficiency can easily lead to swelling and abnormalities in the hoof joints and transverse hoof cracking, causing movement disorders [22,23]. We hypothesized that the decrease in serum Zn and Mn levels in hoof-deformed cows may be related to the increase in Fe levels, and that excess Fe inhibits the absorption, transport, and utilization of Zn and Cu.

### 4.3. Combined Analysis of Differential Metabolites and Differential Ions

In the biosynthesis pathways of valine, leucine, and isoleucine, valine is produced from pyruvate through a number of steps [24]. Valine enters the organism and is rapidly and actively absorbed in the small intestine through the Na^+^-amino acid-carrier complex [25]. The absorption of the gluconeogenic product α-D-glucose also requires the synergistic effect of Na ions [26]. Decreased Na ion levels lead to impaired valine metabolism in the small intestinal mucosa, whereas in skeletal muscle metabolism, skeletal muscle is the main site of the transamination of branched-chain amino acids [27]. Thus, L-valine levels are elevated and biosynthesis pathways of valine, leucine, and isoleucine are altered. In animals, Ca is mainly present in the form of inorganic salts. Ca ions are essential for normal excitation-contraction coupling in skeletal and cardiac muscle [28]. In this study, there is a positive correlation between calcium ion and L-valine, and it may be that the motor function is affected when the cow’s hoof is deformed, and the muscle consumes more energy after exercise, so the skeletal muscle needs branched-chain amino acids and calcium ion to supplement energy. Therefore, in this study, the calcium ion and L-valine of T group dairy cows increased at the same time. It is currently believed that TPPP is related to membrane Na channels; under TPPP deficiency conditions, the osmotic gradient cannot be maintained, causing the transfer of electrolytes and water [29]. The thiamine content was significantly higher and the Na ion content was significantly lower in the T group in the present study. Presumably, thiamine metabolism is activated when hoof deformation occurs. While Cd can alter thiamine metabolism, resulting in impaired energy metabolism, Mn promotes thiamine storage in the liver. Thiamine also prevents intracellular Pb accumulation, especially in the kidney, liver, and nerve tissues, and Pb poisoning. Therefore, the thiamine level was significantly increased while the Pb level was decreased in hoof-deformed cows; Pb and thiamine were negatively correlated. Pyruvate kinase [30] (PK), also known as adenosine triphosphate pyruvate 2-O phosphotransferase, is one of the three rate-limiting enzymes that regulate glycolysis. While metal ions such as K, Na, Mg, Cu, Zn, and Fe can act as agonists of the enzyme [31,32,33], Nowak et al. [34] showed that potassium ion is an agonist of pyruvate kinase, which increases pyruvate kinase activity, accelerates the production of pyruvate, and then quickly converts it into L-valine. In thiamine metabolism, tyrosine produced through tyrosine metabolism and pyruvate produced through glycolysis are used to produce aminoacetic acid and, finally, thiamine. Serum K ion levels were significantly elevated in hoof-deformed cows, and K ions were negatively correlated with L-valine and positively correlated with L-tyrosine. It is hypothesized that elevated K levels alter multiple pathways, including valine, leucine, and isoleucine biosynthesis, tyrosine metabolism, pyruvate metabolism, and gluconeogenesis.

## 5. Conclusions

The LC-MS/MS results showed that there were 127 differential metabolites between group C and group T, 81 of which were upregulated and 46 of which were downregulated in group T. KEGG pathway enrichment analysis showed that the 127 differential metabolites were mainly enriched in the metabolic pathways of “phenylalanine, tyrosine, and tryptophan biosynthesis”, “tyrosine metabolism”, and “thiamine metabolism”. The ionomic ICP-OES/MS results showed a significant upregulation of 13 ions, including K, Li, Cu, and Na, in the serum of group T cows. The levels of Na and 18 other ions were significantly decreased. K ions were positively correlated with L-tyrosine and L-proline, positively correlated with thiamine, and negatively correlated with L-valine; Na ions were positively correlated with L-valine and negatively correlated with α-D-glucose. This study showed significant differences in serum metabolites and ions in hoof-deformed cows compared to healthy cows. These differences may be closely related to hoof deformation.

## Figures and Tables

**Table 1:** Differential metabolites.

Number	MS2 Name	VIP	*p*-Value	Trend
1	D-Pipecolic acid	2.668699	0.000349	↑
2	PI(18:1(9Z)/18:1(9Z))	2.654024	0.000005	↑
3	PI(20:2(11Z,14Z)/16:0)	2.597098	0.000009	↑
4	(+)-2,3-Dihydro-3-methyl-1H-pyrrole	2.477785	0.000858	↑
5	2-Pyrocatechuic acid	2.445668	0.000505	↑
6	PC(16:0/16:0)	2.408921	0.000161	↑
7	Tetradecanedioic acid	2.339200	0.000255	↑
8	PC(18:0/18:2(9Z,12Z))	2.332310	0.000042	↑
9	L-Pipecolic acid	2.300073	0.000196	↑
10	SM(d18:1/16:0)	2.242649	0.000440	↑
11	N6-Methyladenosine	2.238746	0.000207	↑
12	Phenylalanyl-Tryptophan	2.232094	0.000812	↑
13	SM(d18:0/18:1(9Z))	2.228645	0.005772	↑
14	PE(16:0/18:2(9Z,12Z))	2.220292	0.003756	↑
15	Pyro-L-glutaminyl-L-glutamine	2.217429	0.003785	↑
16	PI(20:3(5Z,8Z,11Z)/16:0)	2.199342	0.000667	↑
17	Salicyluric acid	2.197370	0.003655	↑
18	L-alpha-Amino-1H-pyrrole-1-hexanoic acid	2.175090	0.002922	↑
19	p-Aminobenzoic acid	2.145768	0.003594	↑
20	1,2,5,6-Tetrahydro-4H-pyrrolo [3,2,1-ij] quinolin-4-one	2.122660	0.012241	↑
21	Caffeine	2.109282	0.000178	↑
22	MG(0:0/18:3(6Z,9Z,12Z)/0:0)	2.101451	0.018738	↑
23	Polyoxyethylene (600) monoricinoleate	2.093821	0.001234	↑
24	PC(16:0/P-16:0)	2.051576	0.001384	↑
25	Isolithocholic acid	2.016556	0.016698	↑
26	2-Acetyl-3-ethylpyrazine	1.947635	0.002446	↑
27	Glyceraldehyde	1.908274	0.006012	↑
28	L-Targinine	1.854960	0.011832	↑
29	SM(d16:1/24:1(15Z))	1.849228	0.012706	↑
30	9,10-DHOME	1.836305	0.005436	↑
31	5-Aminopentanal	1.829376	0.011143	↑
32	PC(20:2(11Z,14Z)/14:0)	1.808546	0.008800	↑
33	PC(18:2(9Z,12Z)/18:0)	1.781874	0.001949	↑
34	Gamma-Aminobutyric acid	1.768406	0.008607	↑
35	Cholesta-4,6-dien-3-one	1.750652	0.012313	↑
36	Thiamine	1.724694	0.013492	↑
37	LysoPE(20:4(8Z,11Z,14Z,17Z)/0:0)	1.718007	0.010184	↑
38	SM(d17:1/24:1(15Z))	1.716706	0.010800	↑
39	Gamma-Linolenic acid	1.713555	0.029126	↑
40	L-cis-4-(Hydroxymethyl)-2-pyrrolidinecarboxylic acid	1.708745	0.019724	↑
41	Allantoin	1.707810	0.021897	↑
42	Alpha-D-Glucose	1.678138	0.021855	↑
43	D-Proline	1.677953	0.020564	↑
44	PC(20:2(11Z,14Z)/22:0)	1.673556	0.017954	↑
45	N-methylvaline	1.672496	0.008310	↑
46	Gyromitrin	1.625696	0.008534	↑
47	Deoxyadenosine	1.620021	0.012983	↑
48	Histidinyl-Tryptophan	1.604395	0.027128	↑
49	Norvaline	1.603496	0.012239	↑
50	Dihydrouracil	1.588725	0.017140	↑
51	L-Proline	1.573870	0.039936	↑
52	Isoliquiritigenin	1.569349	0.024356	↑
53	PC(18:0/14:0)	1.569301	0.022090	↑
54	4-Acetylimidazo [4,5-c]pyridine	1.561847	0.011886	↑
55	LysoPC(10:0)	1.548761	0.028694	↑
56	L-Norleucine	1.546556	0.016193	↑
57	LysoPE(18:2(9Z,12Z)/0:0)	1.545948	0.025037	↑
58	Benzaldehyde	1.540118	0.026410	↑
59	PC(18:0/P-18:1(11Z))	1.533916	0.031740	↑
60	PC(22:2(13Z,16Z)/15:0)	1.527947	0.023486	↑
61	Histidinyl-Serine	1.524589	0.036587	↑
62	3,3,5-triiodo-L-thyronine-beta-D-glucuronoside	1.510032	0.024113	↑
63	L-Tyrosine	1.509295	0.015867	↑
64	Sorbitol	1.508294	0.028089	↑
65	Arborinine	1.505868	0.023651	↑
66	Phenol glucuronide	1.471716	0.030701	↑
67	L-Phenylalanine	1.437564	0.035461	↑
68	PC-M6	1.428780	0.020437	↑
69	3-Amino-2-piperidone	1.427910	0.022974	↑
70	Glutaric acid	1.426541	0.037374	↑
71	1-Pyrroline-5-carboxylic acid	1.421023	0.040679	↑
72	PC(22:1(13Z)/15:0)	1.395801	0.042589	↑
73	Oxoglutaric acid	1.394322	0.048040	↑
74	Dimethyl dialkyl ammonium chloride	1.374757	0.042075	↑
75	Pyruvic acid	1.374220	0.037639	↑
76	Indole-3-propionic acid	1.369619	0.040906	↑
77	p-Isopropylphenol	1.366147	0.035698	↑
78	1H-Indole-3-acetamide	1.349611	0.044769	↑
79	6-Chloro-N-(1-methylethyl)-1,3,5-triazine-2,4-diamine	1.278744	0.021874	↑
80	(Â±)-Tryptophan	1.238873	0.026068	↑
81	Polyethylene, oxidized	1.172357	0.019404	↑
82	o-Cresol	1.807837	0.014683	↓
83	(9S,10E,12Z,15Z)-9-Hydroxy-10,12,15-octadecatrienoic acid	1.339107	0.038038	↓
84	Docosatrienoic acid	1.352341	0.011775	↓
85	PC(22:5(7Z,10Z,13Z,16Z,19Z)/20:4(5Z,8Z,11Z,14Z))	1.359588	0.037668	↓
86	PC(22:6(4Z,7Z,10Z,13Z,16Z,19Z)/18:1(11Z))	1.396304	0.042004	↓
87	1,3-Dimethyluric acid	1.451010	0.006453	↓
88	PC(18:3(6Z,9Z,12Z)/P-18:1(11Z))	1.461901	0.049885	↓
89	PC(18:1(11Z)/14:0)	1.497574	0.013564	↓
90	Epinephrine	1.506148	0.025789	↓
91	L-Valine	1.545543	0.032929	↓
92	Squamolone	1.548931	0.035178	↓
93	6-Hydroxy-5-methoxyindole glucuronide	1.564298	0.037996	↓
94	2-Piperidinone	1.569621	0.021916	↓
95	Gentisic acid	1.584450	0.025065	↓
96	3-Hydroxyisovaleric acid	1.614573	0.014063	↓
97	Hippuric acid	1.635571	0.019059	↓
98	Pentadecanoic acid	1.644352	0.014762	↓
99	(10E,12Z)-(9S)-9-Hydroperoxyoctadeca-10,12-dienoic acid	1.647604	0.015996	↓
100	Creatinine	1.702533	0.018042	↓
101	Succinic acid semialdehyde	1.712312	0.024760	↓
102	Benzyl acetate	1.798529	0.015313	↓
103	12-Methyltridecanoic acid	1.831773	0.021880	↓
104	Ethyl dodecanoate	1.836277	0.025608	↓
105	PC(22:5(7Z,10Z,13Z,16Z,19Z)/18:3(6Z,9Z,12Z))	1.838133	0.005631	↓
106	LysoPE(0:0/22:4(7Z,10Z,13Z,16Z))	1.855091	0.007737	↓
107	LysoPC(18:1(9Z))	1.873862	0.004356	↓
108	€-3-Hydroxybutyric acid	1.898526	0.005329	↓
109	2-Hydroxy-3-methylbutyric acid	1.901942	0.005243	↓
110	PC(18:4(6Z,9Z,12Z,15Z)/P-18:1(11Z))	1.920975	0.001968	↓
111	Methylguanidine	1.940260	0.002589	↓
112	PC(20:0/14:0)	1.981505	0.003086	↓
113	Isobutyric acid	1.988003	0.015340	↓
114	4-Acetyl-2(3H)-benzoxazolone	2.019812	0.000882	↓
115	LysoPC(22:6(4Z,7Z,10Z,13Z,16Z,19Z))	2.027527	0.004787	↓
116	Ecgonine	2.032427	0.001834	↓
117	LysoPC(20:4(5Z,8Z,11Z,14Z))	2.035657	0.004187	↓
118	3-Methoxybenzenepropanoic acid	2.114186	0.000912	↓
119	(E)-8-Hydroxy-2-octene-4,6-diynoic acid	2.213267	0.000723	↓
120	Saccharin	2.215125	0.003720	↓
121	Carnosic acid	2.242463	0.000932	↓
122	LysoPC(16:1(9Z)/0:0)	2.246300	0.000373	↓
123	Cortisone	2.363506	0.010462	↓
124	4,6-Dihydroxyquinoline	2.402878	0.000239	↓
125	PC(18:1(11Z)/15:0)	2.450633	0.000058	↓
126	Butylparaben	2.459819	0.000256	↓
127	Diphenylamine	2.575275	0.000218	↓

Note: MS2 name: name of the substance; VIP: the projected importance of the variable obtained from the OPLS-DA model for that substance in that group comparison; *p*-value: the *p*-value obtained from the *t*-test for that substance in that group comparison; Tend: ↓ indicates that the level is lower in group C compared with group T, ↑ indicates that the level is higher in group C compared with group T.

**Table 2:** Differential metabolic pathways.

Differential Metabolites	Metabolic Pathway
L-phenylalanine	Phenylalanine, tyrosine and tryptophan biosynthesis Phenylalanine metabolism
L- Tyrosine	Phenylalanine, tyrosine and tryptophan biosynthesis Phenylalanine metabolism Tyrosine metabolism
Thiamine	Thiamine metabolism
L-Valine	Valine, leucine and isoleucine biosynthesis
Alpha-D-Glucose	Starch and sucrose metabolism Glycolysis or Gluconeogenesis
Pyruvic acid	Valine, leucine and isoleucine biosynthesis Pyruvate metabolism Glycolysis or Gluconeogenesis
Dihydrouracil	beta-Alanine metabolism
Epinephrine	Tyrosine metabolism
Epinephrine	Tyrosine metabolism

**Table 3:** Ion screening results.

id	Compound Name	VIP	*p*-Value	Trend
1	K	1.901603	0.000365	↑
2	Li	1.650740	0.020948	↑
3	Pb	1.319857	0.051836	↑
4	Cd	1.210477	0.071717	↑
5	Ba	1.165220	0.120138	↑
6	Mo	0.942896	0.220352	↑
7	Ti	0.758099	0.223822	↑
8	Co	0.632883	0.407299	↑
9	Cr	0.539478	0.519642	↑
10	Fe	0.572170	0.556518	↑
11	Si	0.716205	0.755569	↑
12	Ca	0.147897	0.834511	↑
13	Se	0.291345	0.960316	↑
14	Al	1.601160	0.001899	↓
15	Cu	1.147357	0.007794	↓
16	Sb	1.488560	0.022543	↓
17	B	1.328378	0.032455	↓
18	Sn	1.238449	0.046691	↓
19	Na	1.413242	0.049185	↓
20	Ag	0.993963	0.099159	↓
21	Mn	1.253383	0.181161	↓
22	Zn	0.790421	0.201062	↓
23	P	1.111163	0.211961	↓
24	Mg	0.700067	0.413513	↓
25	As	0.328768	0.478374	↓
26	Zr	0.212851	0.532474	↓
27	Bi	0.545527	0.679050	↓
28	Sr	0.225083	0.702618	↓
29	Be	0.101009	0.774712	↓
30	S	0.359800	0.924664	↓
31	Ni	0.224000	0.927771	↓

Note: ↓ indicates that the level of group T is lower than that of group C, ↑ indicates that the level of group T is higher than that of group C.

**Table 4:** Target metabolomics verification results.

id	Compound Name	VIP	*p*-Value	Tend
1	L-Proline	1.117977	0.001500	↑
2	L-Phenylalanine	0.959884	0.004450	↑
3	L-Tryptophan	0.910356	0.009355	↑

Note: ↑ indicates that the level is higher in group C compared with group T.

## Data Availability

Data available in the repository between visits can be made public.
